# Is a genome more than its genes?

**DOI:** 10.1007/s00709-022-01744-3

**Published:** 2022-02-16

**Authors:** Peter Nick

**Affiliations:** Botanical Institute, Karlsruhe Institute of Technology, Karlsruhe, Germany

In his book *The Selfish Gene*, Richard Dawkins (1976) suggests a mechanism to explain the evolution of altruistic behaviour. He proposes a competition between different alleles of a gene for successful reproduction. According to this model, organisms are mere vehicles under control of their genes and the alleles of a gene are in a state of mutual, continuous warfare for the highest rate of reproduction. This does not prevent cooperativity to emerge: Genes promoting altruistic behaviours can be positively selected in a population, when the statistical benefit acquired by cooperation exceeds the individual cost for this selfless behaviour. In this perspective, genes act individually in a “selfish” manner (Dawkins defines “selfish” as “being copied by a Darwinian selection process”), and the behaviour of the genome is nothing more than the sum of actions of individual genes. Dawkins pursues here a classical line of thought, where evolutionary theory referred to economics (and vice versa). In a sense, Dawkins idea translates Adam Smith (1776) into population genetics. The individual pursuit for economic success will, consequently, lead to maximal welfare of the entire society. But is a genome just the sum of its genes, or can it act as an entity? If it acted as an entity, it should be able to constrain the “selfish” behaviour of its components for the sake of genomic integrity. Integrity should become, thus, manifest, in situation, where different genomes must cooperate. Three contributions to the current issue provide interesting facets to this problem.

The first contribution by von Well et al. (2022) asks the question: what happens when genetic integrity is challenged by γ-irradiation? Their motivation derives from practical application because ionising radiation is generally thought to impose negative effects, but sometimes has also been reported to stimulate plant development. In fact, in cereals, radiation with doses below 100 Gy (which would correspond to the dose administered to a patient in a usual ablative cancer therapy) can stimulate the development of roots or flag leaves. The authors tested this effect for a variety of *Emmer* wheat (*Triticum turgidum*), an allopolyploid with duplicated genomes from two progenitor species (*Einkorn*, *T. monococcum*, and an unknown wild species of *Triticum*). Their focus was on the behaviour of the nucleoli that are responsible for rRNA transcription and, thus, can serve as a readout for metabolic activity (Glinska et al. 2016). In untreated controls, there are four nucleoli that are fed by specific sites on two chromosomes deriving from the wild ancestor of *Emmer*. When they administer γ-irradiation to imbibed seeds, they see delays in the cell cycle that are probably caused by more time being required to repair the DNA damage caused by the irradiation. However, they can induce additional nucleoli that now derive from a specific chromosome originating from the *Einkorn* ancestor, which are normally silenced by methylation. These additional nucleoli are found in micronuclei, which allows for two conclusions. First, the silencing of nucleolar organising regions (NORs) can be released when parts of the genome are absent. The most likely scenario would be an inhibition of the *Einkorn* NORs by factors located on the genome deriving from the wild ancestor of *Emmer*. Second, all the factors needed for the activation of a NOR are probably present in the immediate neighbourhood. Thus, the inhibition of nucleolar activity is acting over distance, while the activation is local.

The second contribution by Ribeiro et al. (2022) is using karyogenetics to understand evolutionary relationships in Inca lilies (*Alstroemeria*), a very diverse group of lilies that occur in South America and Australia and are also of commercial interest due to their attractive and variable flowers. Two clades of this genus differ strongly with respect to heterochromatin content, indicating that there is active regulation of transcription activity between different chromosomes that are very asymmetric in this genus, making them especially suitable for cytogenetical approaches. The focus of the study was on the Brazilian species *Alstroemeria longistaminea*, which, in addition to its 2 n = 16 chromosomes, harbours an additional B chromosome. This type of chromosomes is thought to derive from hybridisation events and is found in many species. B-chromosomes behave as “genetic parasites” (Camacho et al. 2000), and the “host genome” reacts by silencing, or by shifting the B-chromosomes into heterochromatin. On the other hand, the B-chromosomes undergo extensive changes that help them escape the “genetic immune response” of the host, for instance by accumulating retrotransposons or repetitive elements. The authors visualise three highly abundant retrotransposons that account for 45% of the genome in this species, but they find that these are equally distributed over all chromosomes. Instead, they identify a particular satellite sequence, *AloSAT5*, that highlights the entire B-chromosome, while other satellite sequences also labelled the other chromosomes in a banding pattern. The B-chromosome is also covered by 35S rDNA, interspersed mutually with the *AloSAT5* repeats. When the authors investigated closely related species that lack the B-chromosome, they observed that those satellite sequences revealed a very similar pattern which tells that these species maintain their karyotype in a strict manner. The banding pattern also supports the hypothesis that the B-chromosome derives from a terminal region that had broken off from one of the usual chromosomes. Overall, this study illustrates how a genome actively, by specific and complex mechanisms, maintains its integrity.

The third contribution by Shen et al. (2022) follows the behaviour of mitochondria during the life cycle of a brown alga, *Mutimo cylindricus*. These fascinating organisms have arisen from secondary endosymbiosis between algal and fungal ancestors and show a generation cycle. Like in animals, inheritance of mitochondrial genes passes through the mother. While the authors had shown mitophagy to be partially responsible for the matrilinear inheritance of mitochondrial genes (Shen et al. 2021), they still wanted to understand how the maternal mitochondria are passed on in such a robust manner. By using high-quality, also aesthetically appealing, electron tomography, they can now visualise the dynamic morphological changes of mitochondria through two generations of the life cycle. Quantifications of mitochondrial numbers and volumes strongly suggest that the mitochondria fuse just in time before the female gamete is released, thus using a cell biological process to ensure the integrity of the female mitochondrial genome.

What do the three contributions tell about genomic integrity? The wild B-genome in *Emmer* wheat is silencing the NORs of the *Einkorn* ancestor, and this silencing can be released by creating damage through γ-irradiation (von Well et al. 2022). The genome of *Alstroemeria longistaminea* is silencing the B-chromosome that had broken off from the terminus of one of the other chromosomes (Ribeiro et al. 2022). The mitochondria of the *Mutimo cylindricus* oocyte fuse into one entity to escape the autophagic process that will doom their male counterpart to death. All three cases provide evidence for active maintenance of genomic integrity. How can we envisage this “integrity”? Is it a master template which will highlight and correct deviations? While the integrity of a gene can be secured by such a mechanism because the complementary strands of DNA can serve as template when repair is needed, this would not work for a genome, unless one wants to resort to a metaphysic concept of “genomic identity”. It may be more fruitful to conceive integrity as a process resulting in genomic integrity, rather than to see integrity as a physical (or metaphysical) object. If the factors required for silencing (in case of the *Einkorn* genome or the B-chromosome) or the factors required for autophagy (in case of mitochondrial inheritance) are limited in abundance, recruitment to a particular site would by itself result in the depletion from other sites. Mutual competition for a limited resource would then lead to patterning across the genome without the need of a superimposed “genetic identity”. Are these competitive interactions between different genetic loci the cellular correlate of the “invisible hand” that according to Adam Smith (1773) regulates the commonwealth and emerges from the economic competition between otherwise unrestrained individuals?
